# Characterizing Family Contextual Factors and Relationships with Child Behavior and Sleep Across the Buffering Toxic Stress Consortium

**DOI:** 10.1007/s11121-021-01243-6

**Published:** 2021-05-26

**Authors:** Tiffany Phu, Elly Miles, Amy Dominguez, Jason Hustedt, Sarah Enos Watamura, BTS Consortium Principal Investigators

**Affiliations:** 1 Department of Psychology, University of Denver, Denver, CO USA; 2 Department of Human Development and Family Sciences, University of Delaware, Newark, DE USA

**Keywords:** Early Head Start, Family risk and protective factors, Child behavior, Child sleep

## Abstract

The Buffering Toxic Stress (BTS) consortium included six sites in locations that varied widely in racial/ethnic composition and population density. Each site tested a promising parent–child intervention designed to supplement Early Head Start (EHS) services and prevent “toxic stress.” To better understand family risk in a large and diverse EHS sample, studies gathered extensive data on family risk exposure, including demographic risk factors (single mother, unemployed, less than high school education or its equivalent, and neighborhood safety), income-to-needs ratio, household resource constraints, perceptions of economic hardship and pressure, caregiver mental health, and caregiver-reported dysfunctional parent–child interactions. Results presented here for all six sites offer context for the more targeted studies in this special issue. Average levels of family characteristics and child behavior varied by site. We also characterized associations between family characteristics, observer-rated child temperament, and child outcomes (i.e., caregiver-reported child behavior problems and behavioral sleep quality), controlling for child age; these relationships were similar across sites. Demographic risk and caregiver mental health problems were positively associated with child behavior problems, with low income-to-needs ratio and increased financial strain relating to behavioral problems in infancy and toddlerhood. Caregiver mental health problems, financial strain, and social and affect temperament dimensions were related to increased behavioral sleep problems. Dysfunctional parent–child interactions and household resource constraints did not demonstrate statistically significant associations. Findings suggest helpful targets to increase effectiveness of parent–child interventions in early childhood on behavior and sleep outcomes.

## Introduction

The Buffering Toxic Stress (BTS) consortium was a cohort of studies funded by the Administration for Children and Families (ACF), Office of Planning, Research and Evaluation. Each study in the BTS consortium partnered with Early Head Start (EHS) programs to (1) characterize the construct of toxic stress, (2) test the efficacy of promising parent–child interventions in ameliorating negative effects of stress exposure for EHS families, (3) evaluate intervention implementation requirements and barriers among families facing multiple risks (Buffering Toxic Stress Consortium Principal Investigators et al., 2013). The current study aims to (1) contextualize studies in this special issue and (2) investigate the relationship between family characteristics (e.g., complex risk exposure, caregiver mental health) and child outcomes (e.g., sleep, behavior) across the BTS consortium sites.

## National Studies of Early Head Start and Family Characteristics

EHS is a federally funded program that provides infants and toddlers and their families with comprehensive early childhood education and family support services. It has been rigorously evaluated since its inception by national studies such as the Early Head Start Research and Evaluation Project (EHSREP) and the Early Head Start Family and Child Experiences Survey (Baby FACES). Although different in their scope and purpose, both longitudinal studies provide valuable information regarding the characteristics of families enrolled in EHS, support for the efficacy of EHS (Love et al., 2005), and insight into which families benefit most from these services. Given that all sites in the BTS consortium recruited families enrolled in EHS and tested the efficacy of supplemental interventions, EHSREP and Baby FACES provide crucial background for understanding family characteristics and child outcomes in the BTS consortium.

Prior data examining participation of high-risk families in EHS informs our current focus on family context and child outcomes. For example, EHSREP studies have documented that cumulative family demographic risk (measured as teenage parent, single parent, parent neither employed nor in school, receiving cash assistance, and less than high school education) appears to differentiate patterns of EHS participation and its long-term effects, even within the restricted range of risk among all EHS-qualified families (Raikes et al., 2013). Results from Baby FACES similarly showed that families experiencing medium and higher levels of demographic risk (operationalized identically to EHSREP) were rated as less involved in EHS services than lower demographic risk families; in turn, family involvement predicted better behavioral outcomes in preschool above and beyond family characteristics (Vogel et al., 2015).

Prior work suggests that EHS programming could be adapted for greater efficacy for Latinx families, who overall do not show as strong impacts across parent and child outcomes during or after receiving EHS services compared to White and Black non-Latinx families (Raikes et al., 2013). The BTS cross-site sample extends large-scale EHS research with historically underrepresented communities by including a higher proportion of Latinx families (53%) compared to both EHSREP (24%) and the 2009 Baby FACES study (35%); it also includes American Indian children who are almost entirely absent in the EHSREP and Baby FACES samples (in 2015, the American Indian and Alaska Native Head Start Family and Child Experiences Survey began collecting data to fill this gap). As the BTS consortium intended to test promising interventions to increase EHS program impact, the higher proportion of Latinx families allows us to explore relationships between family characteristics and child outcomes among these families, while the inclusion of American Indian children and families allows for rare representation in studies of EHS and children and families in EHS.

## Linkages Between Family Characteristics and Child Outcomes

Behavioral and sleep problems in early childhood may be particularly important outcomes to attend to, as they can reflect current regulatory capacity and cascade developmentally into later childhood and beyond (e.g., Briggs-Gowan et al., 2006; Williams et al., 2017). While early emerging behavior problems are well recognized as important precursors to overall adjustment, sleep is also an important biobehavioral construct linked to child development across emotion, cognition, and biology (Spruyt, 2019). As parents can strongly influence sleep in early childhood (Sadeh et al., 2010), and as sleep and behavior problems can cooccur and contribute to transactional risk, we highlight these outcomes here.

The Family Stress Model (Conger & Elder, 1994) provides a framework whereby economic conditions impact child outcomes in part via impacts on parent mental health. In Baby FACES, parent psychological risk predicted child behavior problems at age 3 (Vogel et al., 2015). These findings aligned with prior studies showing that the relationship between parent mental health and child outcomes is significant for children of all risk levels. For instance, maternal depressive symptoms predict more behavior problems and lower cognitive skills among children; maternal anxiety, stress, and depression during pregnancy similarly increase risk for poorer child outcomes (Glover, 2014). At initial enrollment in EHS services, almost half of mothers endorse moderate to severe depressive symptoms (Vogel et al., 2015). Importantly, maternal depressive symptoms and parenting stress decline as children age, irrespective of EHS participation, with program impacts on maternal depressive symptoms showing by middle childhood (Vogel et al., 2010), suggesting that EHS may also support positive child outcomes via this path.

Overall, EHS research underscores the importance of bolstering parent mental health, reducing parenting stress, and targeting parenting behaviors as key means to support healthy child development (Roggman et al., 2009). For example, although EHSREP findings have illustrated that parenting stress negatively impacts children’s expressive language, participating in EHS appears to buffer the effect of parenting stress on expressive language for boys and promotes language development regardless of parenting stress for girls (Vallotton et al., 2012). Changes in parent–child interactions among EHS families appear to have long-lasting effects: parents’ engagement in cognitively stimulating play with their toddlers predicted academic achievement at fifth grade (Cook et al., 2011).

## The Promise of Parent–Child Interventions

EHS directly serves children from low-income families to support developmental outcomes in the context of stressors and disadvantages that can accompany low resources. Poverty can include not only resource restriction but also lowered capacity for caregivers to demonstrate sensitive parenting behaviors and environmental toxins—all of which can negatively shape child brain development (Johnson et al., 2016). Parent–child interventions, including those tested within the BTS consortium, may mitigate the effects of experiencing childhood poverty on child outcomes by supporting positive parent–child relationships. Indeed, using the Attachment and Biobehavioral Catchup (ABC) Intervention, one BTS consortium team has demonstrated improvements in child physiological regulation during a standardized stress task (Berlin et al., 2019) and for sensitive parenting behaviors (Berlin et al., 2018). Supporting parents can powerfully reduce the impacts of socioeconomic disparities on child outcomes. Coupled with supports directly targeting disparities, (e.g., education, job training, health and mental health, housing, and cash transfer), parenting and parent–child interventions show incredible promise to interrupt links between adversity exposure and compromised potential (Patel et al., 2016).

## Study Rationale

Here, we aim to build on previous work in the EHSREP and Baby FACES studies by examining cross-site relations among family contextual factors and early emerging child sleep and behavior problems in a racially/ethnically diverse sample of EHS infants and toddlers to (1) provide descriptive information on child and family characteristics, especially complex risk exposure and caregiver mental health, across the BTS consortium sites and (2) characterize how family characteristics relate to behavioral and sleep outcomes in children. We operationalize a demographic risk composite to mirror the construct used in EHSREP and build on previous findings by examining additional family risk factors (e.g., economic pressure, caregiver mental health). This approach is consistent with previous cross-site BTS research showing the importance of examining more nuanced family characteristics beyond family income when predicting caregiver and parenting outcomes (Hurwich-Reiss et al., 2019). Child characteristics, such as temperament, may independently impact behavior and moderate intervention effects (e.g., Corapci, 2008); therefore, we include observer-rated child temperament to illustrate the impact of temperament on behavior and sleep. Descriptive findings across the BTS sites also situate the evaluations of intervention efficacy in this special issue and help frame comparisons to prior EHS research.

## Method

### Participants

Participants included 1047 caregivers (96% female) and their child (50% male, *M*_age_ = 20.37 months, SD_age_ = 9.00) enrolled in EHS. Participant-reported characteristics, including race/ethnicity, language, and parent education, are provided in Table 1.

**Table 1:** Descriptive statistics for categorical variables by site

	Site (%)					
	1	2	3	4	5	6
Child	*n* = 267	*n* = 95	*n* = 133	*n* = 171	*n* = 224	*n* = 145
Sex (female)	53	45	47	42	50	52
Latinx	82	32	51	72	86^a^	11
Caregiver race		*n* = 89	*n* = 123	*n* = 170	*n* = 224	*n* = 145
White	-	27	41	46	12	69
Black	-	0	31	18	9	21
Asian	-	0	2	1	1	1
American Indian/Alaskan Native	-	73	1	1	1	0
Biracial/Multiracial	-	0	6	5	1	10
Other	-	0	20	29	75	0
Language spoken at home	-	*n* = 93	*n* = 136	*n* = 146	-	*n* = 144
Monolingual English	-	75	51	22	-	86
Monolingual Spanish	-	1	28	25	-	0
Monolingual other	-	0	0	1	-	1
Bilingual English/Spanish	-	22	19	45	-	5
Bilingual English/other	-	0	2	7	-	8
Marital status	*n* = 241	*n* = 91	*n* = 136	*n* = 169	*n* = 224	*n* = 145
Single	27	17	39	46	18	41
Married	39	44	25	35	39	28
Living with partner	34	31	29	1	40	24
Divorced	0	7	6	12	0.4	6
Separated	0	1	1	3	2	2
Widowed	0	1	0	3	0	0
Employment	*n* = 239	*n* = 94	*n* = 128	*n* = 170	*n* = 224	*n* = 143
Full-time	17	37	23	19	13	21
Part-time	16	1	16	10	18	15
Unemployed	5	2	6	1	1	2
Looking for work	14	4	7	3	12	11
Student	0	19	0	5	0	1
Keeping house/raising children	49	36	48	62	55	50
Household income	*n* = 251	*n* = 61	*n* = 103	*n* = 165	*n* = 148	*n* = 123
Less than $25,000	88	46	86	67	76	72
$25,000–$49,999	9	34	12	29	22	24
Over $50,000	3	20	2	4	2	5
Household resource constraints	*n* = 246	*n* = 93	*n* = 132	*n* = 167	*n* = 223	*n* = 144
Lack access to car	78	3	14	10	-	18
Rent (vs own)	97	76	83	71	-	83
Not enough space	38	10	19	31	32	23
Emergency repairs	8	8	6	19	13	17
Heat/plumbing problems	11	5	7	13	9	13
Problems monthly bills	25	28	41	27	42	66
PC mental health	*n* = 238	*n* = 94	*n* = 133	*n* = 168	*n* = 224	*n* = 136
Depression above clinical cutoff	21	19	26	26	21	31
Anxiety above clinical cutoff	9	12	11	21	8	20

^a^Caregiver ethnicity was used as a proxy for child ethnicity

### Procedure

Participants were recruited across six sites as part of a broader consortium (Buffering Toxic Stress Consortium Principal Investigators et al., 2013) testing promising parent–child relationship interventions for EHS families. The sites included two northeastern cities and surrounding areas, one mid-Atlantic city and surrounding suburban areas, one Midwestern city and surrounding rural area, one western metropolitan area, and one southwestern American Indian community. Here, we focus on baseline data collected prior to intervention delivery. Although the BTS consortium aimed to gather data on several common constructs in a connected set of pilot investigations, each study was conducted independently, and some differences were necessary due to variations in local populations and priorities. Prior to the analyses reported here, data were examined for comparability and converted and combined only where appropriate. Not all measures included in analyses were administered by every site (see Table 1 for details). Variable composites accounted for systematic missingness by calculating percentages, and this is noted where relevant in the section below by measure.

### Measures

#### Demographic Risk Composite

A demographic risk composite variable was created using available measures across sites that mirrored those included in previous work (Love et al., 2005; Vogel et al., 2015) as a percentage of endorsed items among four dichotomously coded risk factors: single mother, less than high school education or equivalent, unemployed, and elevated neighborhood risk. Primary caregivers (PCs) reported their marital status, education, and employment. *Single mother.* “Single” was coded if PCs reported not being married or living with a partner. “Married” included legally married, traditional American Indian marriages, common law marriage, and married spouses living in separate countries. *Less than high school education or equivalent.* Education response options somewhat varied by site; responses to this question were collapsed and recoded for consistency across sites, resulting in five categories: less than high school, some high school, high school diploma or GED, some college or vocational/technical school, and college degree or higher. *Unemployed.* Employment was represented using six categories: full-time work, part-time work, unemployed, looking for work, student, and stay-at-home parent. Unemployment was coded as 1 if not engaged in full- or part-time work.

#### Elevated Neighborhood Risk

In five of the six BTS sites, home visits were conducted. After each home visit, data collectors completed an adapted version of the Windshield Home Observation Survey (Laraia et al., 2006), a measure of perceived neighborhood safety and family preparedness for the visit adapted to achieve brevity and sufficient detail. The adapted Windshield survey consists of 12 multiple-choice questions with response choices from 0 to 4. Six items pertained to family preparedness and warmth (e.g., receptivity toward visitors, ease completing interview), while the other six items pertained to observed safety (e.g., safety of the home’s interior, safety of the building’s exterior, neighborhood safety, neighborhood noise). Elevated neighborhood risk was coded as 1 if the neighborhood observed safety average was 3 or above (range 0–4), with higher average scores indicating lower safety.

#### Economic Hardship and Income

*Income-to-needs ratio.* PCs were asked about family income, which was converted to estimated pre-tax yearly income and reduced to an 11-category variable in increments of $5000 to create a stable equivalent variable across sites (less than $5,000 to over $75,000). An income-to-needs ratio was calculated by dividing family annual income (or midpoint of income category if the site only collected categorical data) by the relevant federal poverty line in the median study year (2013) using the total number of reported adults and children in the household.

#### Household Resource Constraints

PCs responded to dichotomous items assessing lack of access to a car, renting (vs. owning) one’s home, not enough space, household needing emergency repairs, difficulty paying monthly bills, and household heating issues. Household resource constraints were indexed using a percentage of endorsed items across these six items.

#### Perceptions of Economic Hardship and Pressure

PCs completed a 4-item economic hardship and pressure questionnaire adapted from Conger and Elder Jr. (1994). Economic hardship questions assessed whether the family had been without phone services, unable to pay rent or mortgage, evicted, or unable to pay the utility bill within the past year. One site did not ask about utility bills; a percentage was calculated of potential items endorsed. Perceptions of economic pressure were captured by two questions assessing difficulty paying monthly bills and amount of money left at the end of each month. Economic pressure and economic hardship were *Z*-scored and summed to create a composite score of perceived economic difficulties.

#### Caregiver Mental Health

PCs reported on current depressive symptoms using the Center for Epidemiological Studies Depression Scale (CES-D; Radloff, 1977), which consists of 20 items about symptom frequency over the past week, ranging from (0) *rarely or none of the time* to (3) *most or all of the time*. The current study used a total sum of symptoms, which could range from 0 to 60. This well-established measure has been widely utilized in research studies, demonstrating good reliability among ethnically diverse caregivers (*α* = 0.90–93; Beeber et al., 2010) and in the current sample (*α* = 0.88). PCs also reported on current anxiety symptoms via the Generalized Anxiety Disorder 7-item scale (GAD-7, *α* = 0.92; Spitzer et al., 2006), which asks the extent to which PCs were bothered by symptoms (e.g., feeling nervous, anxious, or on edge and not being able to stop or control worrying) over the past 2 weeks. Responses range from (0) *not at all* to (3) *nearly every day.* The current study used a total summary score, which could range from 0 to 21. The GAD-7 demonstrated good reliability in the current sample (*α* = 0.88).

A composite variable indexing caregiver mental health symptoms was created by summing *Z* scores of caregiver depression and anxiety. Depressive and anxiety symptoms were also characterized using each measure’s clinical cutoff to aid interpretation in the discussion section, where clinical cutoffs include scores of 16 or above for CES-D (Radloff, 1977) and 10 or more for the GAD-7 (Spitzer et al., 2006).

#### Parent–Child Dysfunctional Interaction

The Parenting Stress Index-Short Form (PSI-SF; Abidin, 1990) consists of 36 items. Response choices range from (1) *strongly agree* to (5) *strongly disagree*, with higher scores indicating greater parenting stress. Only the Parent–Child Dysfunctional Interaction subscale, a sum of 12 items, was used across all sites and included in analyses. The PSI-SF has been validated across all subscales among caregivers experiencing low-income levels (*α* = 0.76; Whiteside-Mansell et al., 2007). The PSI-SF Parent–Child Dysfunctional Interaction subscale demonstrated good reliability in the current sample (*α* = 0.81).

#### Child Behavior Problems

The Brief Infant–Toddler Social and Emotional Assessment (BITSEA; Briggs-Gowan et al., 2002) is a 42-item measure assessing social and emotional competence among 2- to 36-month-olds. PCs completed the BITSEA. One site used the full 166-item Infant–Toddler Social and Emotional Assessment (Carter & Briggs-Gowan, 2000) and extracted all BITSEA items and scales for the current analyses. The BITSEA yields two scales: Problems and Competence (*α*s > 0.70; Briggs-Gowan & Carter, 2007). Preliminary evidence indicated adequate to low reliability for these scales (*α*s = 0.85 and 0.57) within low-income, Hispanic 12–18-month-olds (Hungerford et al., 2015). In the BTS sample, BITSEA internal consistency across all six sites was 0.76. Two sites also asked PCs complete the Child Behavior Checklist (CBCL; Achenbach & Rescorla, 2000) for children older than 18 months. The CBCL is a widely used, standardized, parent report measure that evaluates maladaptive behavioral and emotional problems, assessing internalizing and externalizing domains. Caregivers are asked to indicate how characteristic each problematic behavior has been for the child in the last 2 months (0 = not true to 2 = very true). The CBCL has shown good internal consistency elsewhere (*α* = 0.76–0.88; Rescorla, 2005) and in this sample (*α* = 0.95). BITSEA problems and CBCL problem behaviors were made comparable by creating an overall percentage of endorsed problems, which consisted of a sum across item ratings divided by the total possible score for internalizing and externalizing problems in each scale. For descriptive purposes, a dichotomous variable indicating clinically significant problems (across internalizing and externalizing) was calculated using BITSEA cutoff problem scores by child age and sex (Briggs-Gowan et al., 2002) and CBCL Total Problem *T* scores of 65 and above.

#### Child Sleep

PCs reported on behavioral sleep quality using three subscales (going to bed, falling asleep at night, and falling asleep for naps) from the Child Sleep–Wake Scale (LeBourgeois & Harsh, 2016). One site used slightly different wording for four items asking about number of naps, bedtime, wake time, and nighttime awakenings. The CSWS was normed within a majority-white, low-, and middle-income sample of 2 to 8-year-old children (*α'*s = 0.81–0.91; LeBourgeois & Harsh, 2016). Internal consistency in the BTS sample was 0.71–0.74.

#### Other Demographics

Child age was calculated by subtracting child birth date from initial study visit date (see Table 2). Caregivers identified caregiver and child race using a broad set of response options, which were subsequently collapsed based on frequency into the following categories: White, Black, Asian, American Indian/Alaska Native, Biracial/Multiracial, and other. Caregivers also reported on their own and their child’s ethnicity (Hispanic/Latinx yes/no). Languages spoken in the home were recoded using questions assessing English capabilities (*not at all* to *very well*) and other language spoken in the home (*Spanish* or *other*). Low English capability (*not at all, not well*) and Spanish spoken in home were coded as Monolingual Spanish, whereas high English capability (*well, very well*) and Spanish spoken in home were coded as bilingual English/Spanish. Two sites did not assess English capability and have missing language data. See Table 1 for details.

**Table 2:** Descriptive statistics for continuous variables by site

	Site, *M* (SD)					
	1	2	3	4	5	6
Child age (months)	(*n* = 267) 23.89 (8.38)	(*n* = 88) 16.68 (4.59)	(*n* = 102) 20.38 (9.47)	(*n* = 170) 23.98 (9.30)	(*n* = 224) 13.03 (4.04)	(*n* = 145) 23.21 (9.42)
Economic hardship	(*n* = 243) 1.16 (1.26)	(*n* = 94) 0.69 (1.07)	(*n* = 99) 1.02 (1.19)	(*n* = 170) 0.77 (1.09)	(*n* = 224) 0.64 (0.97)	(*n* = 145) 1.41 (1.19)
Neighborhood characteristics						
Observed safety	(*n* = 240) 2.93 (.39)	-	(*n* = 92) 2.73 (.60)	(*n* = 170) 2.69 (.43)	(*n* = 220) 3.03 (.18)	-
Family preparedness/warmth	(*n* = 245) 3.09 (.48)	-	(*n* = 94) 3.34(.47)	(*n* = 170) 2.72(.44)	(*n* = 222) 3.22 (.33)	-
PC mental health						
PC depression	(*n* = 238) 10.95 (9.00)	(*n* = 94) 9.53 (7.60)	(*n* = 133) 10.87 (9.49)	(*n* = 169) 11.73 (10.11)	(*n* = 224) 9.22 (8.99)	(*n* = 136) 12.52 (9.52)
PC anxiety	(*n* = 236) 3.77 (4.05)	(*n* = 94) 3.78 (4.45)	(*n* = 137) 3.97 (4.91)	(*n* = 169) 5.59 (4.87)	(*n* = 224) 3.41 (4.08)	(*n* = 122) 6.12 (4.95)
Child temperament						
Social approach	(*n* = 252) 12.87 (2.58)	-	(*n* = 99) 14.20 (2.17)	(*n* = 165) 13.45 (2.68)	(*n* = 222) 15.55 (1.35)	(*n* = 119) 13.65 (2.45)
Affect	(*n* = 252) 12.30 (3.48)	-	(*n* = 99) 13.60 (2.43)	(*n* = 165) 12.13 (3.42)	(*n* = 222) 13.00 (2.07)	(*n* = 119) 13.15 (2.33)
Attention	(*n* = 252) 17.62 (4.10)	-	(*n* = 99) 18.18 (2.61)	(*n* = 165) 14.32 (4.85)	(*n* = 222) 18.30 (2.56)	(*n* = 119) 16.89 (3.53)
Child sleep						
Going to bed	-	(*n* = 24) 10.54 (3.89)	-	(*n* = 92) 9.71 (3.58)	-	(*n* = 70) 9.40 (4.17)
Falling asleep (night)	(*n* = 176) 14.44 (3.98)	(*n* = 89) 14.10 (4.76)	-	(*n* = 167) 15.19 (3.87)	(*n* = 224) 15.02 (4.52)	(*n* = 139) 15.07 (4.58)
Falling asleep (nap)	-	(*n* = 89) 13.07 (4.98)	-	(*n* = 160) 15.78 (4.19)	(*n* = 224) 15.58 (4.29)	(*n* = 134) 15.29 (4.34)
Child behavior problems (BITSEA/CBCL percentage endorsed)						
Internalizing	(*n* = 188) 21.50 (15.51)	(*n* = 79) 14.50 (10.84)	(*n* = 103) 16.21 (13.62)	(*n* = 135) 18.07 (15.34)	(*n* = 181) 19.92 (13.56)	(*n* = 90) 10.95 (8.98)
Externalizing	(*n* = 188) 18.94 (16.30)	(*n* = 79) 18.00 (16.17)	(*n* = = 103) 15.95 (18.50)	(*n* = 135) 34.79 (21.65)	(*n* = 181) 18.46 (18.12)	(*n* = 90) 21.98 (16.31)

*PC* primary caregiver

#### Observer-Rated Child Temperament

Child temperament was indexed using the Bayley Infant Behavior Record (IBR; Matheny, 1983), an observer-rated measure of infant temperament designed to yield three factors: social approach, affect, and attention. The factor structure of the IBR was established with a sample representative of Louisville, KY, and has been validated within low-income, Black families (Kaplan-Estrin et al., 1994). However, the factor structure did not replicate in our sample, where primarily one factor emerged with a second factor including a rating of the child’s responsivity to their caregiver. Trained research assistants used the IBR to rate in-session child temperament, with some sites averaging across two raters and one site consensus scoring. One site did not administer the IBR. In this sample, internal consistency for temperament factors was acceptable (affect, *α* = 0.71; attention, *α* = 0.83) to poor (social approach, *α* = 0.59). Due to limited tools available for infants and toddlers to assess social approach, we elected to use the IBR understanding these limitations.

### Data Analytic Plan

Descriptive statistics are presented by site to (1) increase understanding of child and family characteristics in the BTS consortium. Multilevel regression models were run in M*Plus*, Version 8 (Muthén, 2018) to test (2) relations between family characteristics (i.e., demographic risk factors, income-to-needs ratio, household resource constraints, caregiver mental health, and PC-reported dysfunctional interactions), observer-rated infant temperament, and child outcomes (i.e., PC-reported problem behavior and sleep quality), controlling for child age. Children were nested within sites. We used full information maximum likelihood for missing data (Dong & Peng, 2013).

## Results

### Descriptive Results

Table 1 provides descriptive statistics for categorical variables by site. Cross-site sample characteristics uniformly indicated a high percentage of families experiencing poverty, and a sizable proportion experiencing deep poverty, with 46–88% of families earning $25,000/year or less. Sites varied more widely on child ethnicity (range 11–82% Latinx). Table 2 provides descriptive statistics for continuous variables by site, including neighborhood characteristics, perceived economic hardship and pressure, PC mental health symptoms (i.e., depression and anxiety), observer-rated child temperament, child sleep, and child behavior problems. When examining total behavior problems using measure cutoffs, 28% of the sample fall in the clinically elevated range.

Analyses of variance demonstrated site-level differences in family characteristics: demographic risk, *F* = 6.54, *p* < 0.001; income-to-needs ratio, *F* = 32.28, *p* < 0.001; household resource constraints, *F* = 39.09, *p* < 0.001; perceived economic hardship, *F* = 13.06, *p* < 0.001; perceived economic pressure, *F* = 24.81, *p* < 0.001; caregiver mental health (i.e., depression, *F* = 3.00, *p* = 0.01; anxiety, *F* = 9.73, *p* < 0.001); parent–child dysfunctional interaction, *F* = 656.61, *p* < 0.001. Sites differed in child behavior problems (i.e., internalizing, *F* = 9.29, *p* < 0.001; externalizing, *F* = 19.00, *p* < 0.001), but not child nighttime sleep (i.e., falling asleep at night, *F* = 1.51, *p* = 0.20; go to bed, *F* = 0.79, *p* = 0.46). Despite site differences, in multilevel models, site accounted for a small amount of variance across child outcomes (see Table 3); however, this level was retained due to methodological and population differences.

**Table 3:** Multilevel regressions (nested within site) predicting child problem behavior from family and child characteristics controlling for child age, using full information maximum likelihood

	Combined BITSEA/CBCL				BITSEA				Sleep quality					
	Internalizing		Externalizing		Competence		Problem		Night		Nap		Go to Bed	
	*β* (b)	t/SE	*β* (b)	t/SE	*β* (b)	t/SE	*β* (b)	t/SE	*β* (b)	t/SE	*β* (b)	t/SE	β (b)	t/SE
Family demographic context														
Demographic risk	0.11^**^ (6.56)	2.94/0.04	0.08^+^ (6.16)	1.91/0.04	− 0.09^***^ (− 1.45)	− 3.83/0.02	0.04 (1.19)	1.00/0.04	0.01 (0.19)	0.40/ 0.03	0.05 (0.94)	1.20/ 0.04	0.01 (0.12)	0.14/ 0.05
Income-to-needs ratio	− 0.04 (− 0.70)	− 0.73/0.05	0.01 (0.11)	0.14/0.03	0.08^***^ (0.37)	3.61/0.02	− 0.11^***^ (− 0.97)	− 2.93/0.02	− 0.08^+^ (− 0.43)	− 1.71/0.05	− 0.15^*^ (− 0.85)	− 2.49/ 0.06	0.06 (0.30)	1.50/ 0.04
Economic hardship and pressure	− 0.04 (− 0.22)	− 0.66/0.04	− 0.05 (− 0.59)	− 1.62/0.03	0.09^**^ (0.20)	3.11/0.03	− 0.06^**^ (− 0.26)	− 2.93/0.02	− 0.03 (− 0.09)	− 1.50/ 0.02	− 0.02 (− 0.04)	− 0.46/ 0.04	− 0.06^***^ (− 0.14)	− 6.16/ 0.01
Household resource constraints	0.08^+^ (5.00)	1.87/0.04	0.04 (2.98)	0.96/0.04	− 0.04 (− 0.60)	− 0.65/0.06	0.08 (2.71)	1.55/0.05	0.01 (0.26)	0.37/ 0.04	0.03^+^ (0.67)	1.80/ 0.02	0.02 (0.43)	0.35/ 0.07
Caregiver mental health and parent–child interaction														
PC mental health	0.07^+^ (0.50)	1.80/0.28	0.26^***^ (2.67)	4.32/0.06	− 0.15^***^ (− 0.29)	− 8.22/0.02	0.31^***^ (1.20)	12.25/0.03	− 0.18^**^ (− 0.42)	− 2.86/ 0.06	− 0.17^+^ (− 0.40)	− 1.96/ 0.08	− 0.00 (− 0.00)	− 0.06/ 0.03
Dysfunctional parent–child interaction	− 0.03 (− 0.04)	− 0.89/0.04	− 0.04 (− 0.06)	− 0.49/0.08	− 0.01 (− 0.00)	− 0.31/0.03	− 0.06 (− 0.04)	− 0.70/0.08	− 0.05 (− 0.02)	− 0.78/ 0.07	− 0.07 (− 0.03)	− 1.04/ 0.07	− 0.05 (− 0.02)	− 0.48/ 0.10
Temperament														
Social	− 0.03 (0.19)	− 0.73/0.05	0.09 (0.72)	1.50/0.06	0.35^***^ (0.51)	4.08/0.09	0.11^***^ (0.33)	3.83/0.03	0.07^+^ (0.11)	1.93/ 0.03	0.07 (0.12)	0.93/ 0.07	− 0.24^***^ (− 0.38)	− 6.48/ 0.04
Affect	− 0.14^**^ (− 0.66)	− 2.86/0.05	− 0.02 (− 0.16)	− 0.45/ 0.05	− 0.20^***^ (− 0.25)	− 3.98/0.05	− 0.18^**^ (− 0.44)	− 3.28/0.05	0.10^**^ (0.15)	3.05/ 0.03	0.09^**^ (0.14)	2.72/ 0.03	0.16^***^ (0.21)	5.06/ 0.03
Attention	0.05 (0.16)	0.98/0.05	− 0.19^***^ (− 0.92)	− 4.06/0.05	0.11^+^ (0.10)	1.72/0.06	− 0.02 (− 0.04)	− 0.36/0.06	− 0.09 (− 0.10)	− 1.13/ 0.08	− 0.04 (− 0.05)	− 0.95/ 0.04	− 0.04 (− 0.04)	− 0.95/ 0.04
*ICC*	*5%*		*10%*		*5%*		*10%*		*0.004%*		*0.49%*		*0.004%*	

*PC* primary caregiver

^*^p < 0.05; **p < 0.01; ***p < 0.001; + *p* < 0.1

### Multilevel Linear Models

As shown in Table 3, several different patterns emerged between family characteristics and child behavioral outcomes. Results showed that child internalizing behavior across BITSEA and CBCL measures was positively associated with demographic risk factors, *β* = 0.11, *p* = 0.003, PC mental health symptoms, *β* = 0.11, *p* = 0.004, and household resource constraints (i.e., not having a car, not enough space, emergency repairs, heating/plumbing problems, problems paying monthly bills), *β* = 0.08, *p* = 0.07. Child externalizing problems were positively associated with PC mental health symptoms, *β* = 0.24, *p* < 0.001, and demographic risk factors *β* = 0.08, *p* = 0.048. Neither internalizing nor externalizing problems across BITSEA and CBCL measures were associated with dysfunctional parent–child interactions or perceptions of economic hardship and pressure. When restricting analyses only to children with BITSEA reports, internalizing sums of child behavior showed similar patterns as the percentage of endorsed items across the BITSEA and CBCL measures, although lower internalizing problems as measured on the BITSEA was also associated with greater perceptions of economic hardship and pressure, *β* =  − 0.08, *p* < 0.001, and higher income-to-needs ratio, *β* =  − 0.08, *p* < 0.001. Externalizing behavior on the BITSEA was positively associated with household resource constraints, *β* = 0.08, *p* = 0.08, and PC mental health symptoms, *β* = 0.32, *p* < 0.001. Lower levels of PC-reported competence on the BITSEA were associated with higher demographic risk, *β* =  − 0.09, *p* < 0.001, lower income-to-needs ratio, *β* = 0.08, *p* < 0.001, lower perceived economic pressure and hardship, *β* = 0.09, *p* = 0.002, and more PC mental health symptoms, *β* =  − 0.13, *p* < 0.001.

Finally, as shown in Table 3, higher levels of PC mental health problems were associated with lower PC-reported child sleep quality at nighttime, *β* =  − 0.18, *p* = 0.002, and naptime *β* =  − 0.17, *p* = 0.03. Additionally, higher income-to-needs ratio was related to lower child sleep quality at naptime, *β* =  − 0.15, *p* = 0.01. Higher perceived economic pressure was associated with a harder time with going to bed, *β* =  − 0.06, *p* < 0.001. Supplementary mediation analyses did not support dysfunctional parent–child interactions as a mediator for child behavior or sleep (results available from first author).

## Discussion

Families across the BTS consortium sites demonstrated elevated demographic risk and high levels of poverty. These findings suggest that the families characterized as at-risk for lowered participation and diminished long-term effects in EHS (e.g., Raikes et al., 2013) were well represented in the parent–child interventions explored in this issue. This paper aimed to describe how family and child characteristics and their relations varied across the BTS consortium sites to aid interpreting other effects. Few cross-site differences emerged for relations between parent and child factors (e.g., child behavior and sleep problems), despite clear site differences in average levels of examined family characteristics and child behavior. This suggests that relations between family characteristics and child outcomes hold true, even across diverse local contexts and sample characteristics. Similarly, several factors suggest the intended target population was reached across consortium sites. The majority of families experienced high levels of economic disadvantage. PC depression also commonly occurred, with 19–31% of PCs meeting clinical cutoffs for depression across BTS sites—a higher rate than the national prevalence of depression for adults living below the poverty line (16%; Brody et al., 2018). However, while the variance attributable to site was generally low, nearly double the amount of site-level variance was identified for externalizing behavior (10%) compared to internalizing behaviors. This finding may reflect literature demonstrating that community-level differences in poverty or other structural disadvantages can significantly influence the development of youth externalizing behaviors (Shaw & Shelleby, 2014). Furthermore, regional variance may reflect differential levels of community violence or safety. For example, one study of behavioral outcomes in Head Start children identified that community violence predicted greater levels of child externalizing, and this relationship was not moderated by positive parenting (Oravecz et al., 2008). One proposed mechanism linking community safety to child problem behaviors is increased authoritarian, harsh, or aggressive parenting (e.g., Zhang & Anderson, 2010). Therefore, effective strategies for reducing early onset of externalizing symptoms may include targeting community violence and its roots alongside promoting supportive parenting, as the interventions here were funded to do.

Greater site-level variance (10%) was also identified for infant social-emotional competence scores. While the causes for regional variance in levels of BITSEA competence remain unclear, it may be that certain promotive factors exist to a greater degree within some communities. For example, some children may benefit from more close-knit communities, stronger educational opportunities, or community-level interventions that promote positive PC health care utilization and behaviors (as one example, see the Text4baby case study; Evans et al., 2012). Additionally, communities may vary in the degree to which comprehensive early screening and referrals for early intervention are integrated and followed up on, resulting in different rates of early intervention uptake (e.g., Talmi et al., 2014). Differences in state-specific early intervention policies and resources may also contribute.

A second goal of this study was to examine how family characteristics are linked with child behavior and sleep outcomes. As expected, higher levels of demographic risk (i.e., single mother, less than high school education or its equivalent, and elevated neighborhood risk) were related to a variety of negative child outcomes. Specifically, demographic risk was linked with greater child internalizing and externalizing symptoms and decreased social-emotional competencies in infancy. As almost a third of the sample demonstrates clinically elevated behavioral problems, these results warrant attention. Perhaps surprisingly, higher income-to-needs ratio was associated with more internalizing symptoms as measured on the BITSEA for infants under 18 months. However, this finding was not replicated within the CBCL only or the combined BITSEA/CBCL data. Given the difficulty assessing very early mental health symptoms, we are not offering an interpretation. Greater economic hardship and pressure was related to more child behavioral problems on the BITSEA but not the combined BITSEA/CBCL, suggesting that financial struggles with utilities and services can especially impact early childhood. Broadly, findings align with literature emphasizing how poverty can expose young children to stressors that directly (Yoshikawa et al., 2012) and indirectly affect child behavior via relational mediators, such as lower quality parenting, and institutional mediators, such as lower quality ECE settings (NICHD Early Child Care Research Network, 2005).

In our sample, PC mental health was linked with early childhood externalizing symptoms and infant problem behaviors. Findings in the BTS sample reflect broader work demonstrating a robust relationship between parent depression and anxiety and child behavioral health symptoms (Glover, 2014). Unexpectedly, dysfunctional parent–child interactions as reported by PCs were not associated with child internalizing or externalizing behaviors, nor did they appear to mediate relationships between family characteristics and child outcomes in supplementary analyses. Given that some research supports linkages from observed parent–child interaction dysfunction to teacher-reported externalizing and internalizing on the CBCL (Hollenstein et al., 2004), it may be that PC report data failed to capture this phenomenon and that direct observation would yield different results. It may also be that dysfunctional parent–child interactions more strongly predict outcomes that were unmeasured here but found in EHSREP analysis, such as maltreatment risk (Green et al., 2020).

Greater PC mental health symptoms were related to greater child difficulties with falling asleep at nighttime and naptime. These findings align with a broader literature highlighting interrelations among caregiver mental health symptoms, parenting behavior, and infant sleep quality (Sadeh et al., 2010). However, the pathways by which parent mental health and child sleep may be related are less clear. Child affect, a temperament factor, was similarly associated with all measures of child sleep quality: infants with more negative affect in our sample had lower night-time sleep quality and greater difficulty with falling asleep at both naptime and bedtime. Given that difficult infant temperament has been associated with both worse sleep quality and maternal mental health symptoms in previous work (e.g., Weinraub et al., 2012), the interactions between these factors warrant ongoing attention. Nonetheless, targeting infant sleep may be one mechanism by which parenting interventions, including those studied within the BTS consortium, affect positive child outcomes. Indeed, sleep is an important predictor and correlate of social, emotional, and cognitive development in early childhood (Astill et al., 2012), supporting its primacy as a child outcome of interest for EHS.

The current study situates innovative parenting interventions examined in the Buffering Toxic Stress Consortium and this special issue within the local and national context by describing child and family characteristics across sites. As one strength for the BTS consortium derives from allowing interventions to respond to local community needs and settings, the populations served by individual sites varied. Attending to family characteristics is important, as families receiving EHS are not homogenous and this heterogeneity occurs in ways that are known to shape program impacts (Raikes et al., 2013). Findings show that within the EHS population, unemployed single parents without a GED or equivalent and living in less safe neighborhoods may be particularly in need of support, as these characteristics were associated with behavior problems independently of variance explained by the effects of experiencing low-income, perceived economic pressure, and dysfunctional parent–child interactions. Parenting interventions working within EHS may need to better attune to these needs, perhaps considering factors targeting stressors related to unemployment, low education, and less safe neighborhoods by reducing time demands, flexible scheduling, reducing unnecessary complexity of information and procedures, and building social capital. Perceived economic pressure and experiencing low income accounted for unique variability in early childhood behavioral problems; access to increased financial resources is likely important in supporting the well-being of EHS families. Diminished caregiver mental health was also associated with increased child behavioral problems and lower nighttime sleep quality, indicating that supporting caregiver mental health may be an important lever of change. Indeed, as these adaptations could likely benefit all parents, this may be a fruitful avenue to pursue.

Limitations of this study include its descriptive, cross-sectional design and inability to tease apart the highly complex relations among family characteristics and experiences and child behavior. Previous longitudinal literature has highlighted transactions among variables that were treated here simply due to the cross-sectional design. For example, the Family Stress Model (Conger & Elder, 1994) underscores the interactive nature of experiences within the family context, including economic hardship, caregiver mental health, marital relationships, and parenting behaviors. Also, sleep quality and duration are known to influence child behavioral problems (Cremone et al., 2018), illustrating testable pathways among child outcomes. Similarly, analyses indicated main effects for observer-rated child temperament and these dimensions (i.e., social, affect, attention) were often significantly associated with child outcomes examined. Interpretation of these findings is limited by poor reliability of the social approach factor. Additionally, given that this rated measure did not factor as designed in this sample, we did not further examine moderation. Future studies should carefully measure child temperament and examine potential interactions with relations among family characteristics and child behavioral and sleep outcomes.
