# Correction: Characterizing Family Contextual Factors and Relationships with Child Behavior and Sleep Across the Buffering Toxic Stress Consortium

**DOI:** 10.1007/s11121-023-01632-z

**Published:** 2024-01-06

**Authors:** Tiffany Phu, Elly Miles, Amy Dominguez, Jason Hustedt, Sarah Enos Watamura, BTS Consortium Principal Investigators

**Affiliations:** 1 Department of Psychology, University of Denver, Denver, CO USA; 2 Department of Human Development and Family Sciences, University of Delaware, Newark, DE USA

**Correction to: Prevention Science** 10.1007/s11121-021-01243-6

The article “Characterizing Family Contextual Factors and Relationships with Child Behavior and Sleep Across the Buffering Toxic Stress Consortium”, written by Phu, T., Miles., E., Dominguez, A., Hustedt, J., Watamura, S.E., and BTS Consortium Principal Investigators, was originally published electronically on the publisher’s internet portal on 26 May 2021 without open access. With the author(s)’ decision to opt for Open Choice the copyright of the article changed on 29 November 2023 to © The Author(s) 2023 and the article is forthwith distributed under a Creative Commons Attribution 4.0 International License, which permits use, sharing, adaptation, distribution and reproduction in any medium or format, as long as you give appropriate credit to the original author(s) and the source, provide a link to the Creative Commons licence, and indicate if changes were made. The images or other third party material in this article are included in the article’s Creative Commons licence, unless indicated otherwise in a credit line to the material. If material is not included in the article’s Creative Commons licence and your intended use is not permitted by statutory regulation or exceeds the permitted use, you will need to obtain permission directly from the copyright holder. To view a copy of this licence, visit http://creativecommons.org/licenses/by/4.0/.

The original article has been corrected.

